# Psychosocial assessment of households covered by health centers, identification of high risk ‎cases and psychological management in crisis and disasters 

**Published:** 2019-08

**Authors:** Haniyeh Rezaei, Tayyebeh Rezaei

**Affiliations:** ^*a*^Tabriz University of Medical Sciences, Tabriz, Iran.

## Abstract

**Background:**

The crisis is the severity and peak of stress. According to the accumulative characteristic of ‎stress, the accumulated stresses become a major crisis over the time. All those exposed to the ‎crisis experience different post-traumatic psychological states. Some people suffer from post-‎traumatic stress syndrome. Some of them suffer from post-traumatic stress due to the ‎observation of dangerous scenes and lose of the beloved ones. Of among, personal vulnerability ‎is one of the most important factors that has a psychologically significant effect on the ‎occurrence or absence of psychological disorders in post-crisis events. According to the ‎importance of this issue, Marand Health Center s Psychology Unit intends to identify people ‎who expose the risk of psychological disorders through a cross-sectional assessment, and ‎develop an operational program required for psychological interventions in crisis and disasters ‎with a prospective approach. ‎

**Methods:**

The present study is a research initiative idea and plan. The total number of households covered ‎by health centers is 77,564 people. To execute this program, it is first necessary to hold ‎meetings with psychiatric centers and clinical experts regarding the purpose and ‎implementation procedure of this program. The evaluation form is then prepared and reviewed ‎according to the psychologists’ perspectives, and its validity and reliability was confirmed by ‎experts. Finally, a training course was held for personnel involved in health centers and required ‎training on how to complete the forms and identify those at risk was presented to them. As well ‎as, in the context of post-incident crisis management (with a prospective approach), follow-up ‎was conducted for those exposed events, and required interventions were provided to prevent ‎the deterioration of the mental condition or the occurrence of psychological disorders. ‎

**Results:**

As event-induced crises on the one hand predispose an individual to psychological disorders ‎and, on the other hand, increase the potential forces and the use of psychological mechanisms, ‎proper intervention on the one hand is therefore beneficial to prevent the immediate symptoms ‎and psychological disorders and on the other hand, it will bring about the individual growth and ‎prosperity. ‎

**Conclusions:**

Since one of the basic discussions in crisis and risk management is the preparation before and ‎the action at the right time, identifying people at risk and training ways to deal with stress can ‎prevent psychological consequences of critical events based on this idea. According to the ‎studies and surveys, the most pre-crisis intervention includes the analysis and readiness of ‎structures, strength creation, and so forth. And there are few studies on the psychological ‎hazards. Therefore, it is suggested that this idea is implemented and evaluated in some ‎disastrous areas of the country.‎

**Keywords:**

Crisis Management, Post-crisis psychological disorders, Post-traumatic stress disorder

